# Editorial: Learned Brain Self-Regulation for Emotional Processing and Attentional Modulation: From Theory to Clinical Applications

**DOI:** 10.3389/fnbeh.2016.00062

**Published:** 2016-03-31

**Authors:** Sergio Ruiz, Niels Birbaumer, Ranganatha Sitaram

**Affiliations:** ^1^Psychiatry Department, Interdisciplinary Center for Neuroscience, School of Medicine, Pontificia Universidad Católica de ChileSantiago, Chile; ^2^Laboratory of Brain-Machine Interfaces and Neuromodulation, Pontificia Universidad Católica de ChileSantiago, Chile; ^3^Institute of Medical Psychology and Behavioral Neurobiology, University of TübingenTübingen, Germany; ^4^Ospedale San Camillo, Istituto di Ricovero e Cura a Carattere ScientificoVenezia, Italy; ^5^Institute for Biological and Medical Engineering, Pontificia Universidad Católica de ChileSantiago, Chile

**Keywords:** neurofeedback, self-regulation, brain-computer interfaces, emotion, attention

Mounting evidence in the last years has demonstrated that self-regulation of brain activity can be achieved by neurofeedback (NF). These methodologies have constituted themselves as new tools for cognitive neuroscience, to establish causal links between volitionally controlled brain activations and cognition and behavior, and as potential clinical applications in severe neuropsychiatric disorders (e.g., schizophrenia, depression, Parkinson's disease, etc.). Current developments of brain imaging-based NF include the study of the behavioral modifications and neural reorganization produced by learned regulation of the activity of circumscribed brain regions and neuronal networks.

In this rapidly developing field, many open questions and controversies have arisen, i.e., choosing the proper experimental design, the adequate use of control conditions and subjects, the mechanism of learning involved in brain self-regulation, and the effects on brain reorganization and clinical alleviation in severe brain disorders, among others. The current research topic includes theoretical, technical and experimental achievements in NF based on EEG/MEG and hemodynamic-based NF.

The first part of the current issue considers several methodological advances in the field.

The growing researcher- and user-base of real-time fMRI-NF applications has prompted a handful of laboratories to build open and free software tools and libraries. Basilio et al. report one such toolbox, named *Functional Real-time Interactive Endogeneous Neuromodulation and Decoding* (FRIEND). The authors present a client-server cross-platform solution, which provides a number of new features including, customization and integration of user-defined graphical interfaces, devices, and data processing, with novel front-ends and plugin support.

Recent studies in NF are increasingly relying on multivariate classification for decoding brain states in real-time for more effective learning. However, an often unrecognized problem is that the classification accuracy is used as a common measure for evaluating the technical performance of a classifier as well as the performance of participants in learning brain self-regulation; thus confounding methodological and neuropsychological outcomes. Bauer and Gharabaghi, propose a solution to this problem based on item-response theory and cognitive load theory, and arrive at a new metric called the zone of maximum development (ZMD) as a measure of participant's cognitive resource and efficacy of NF. Adoption of this measure in future studies should help consolidate its relevance to NF research.

De Massari et al. test the feasibility of online decoding and monitoring of brain states in virtual and mixed reality environments for guiding explicit and implicit learning using ecologically valid scenarios. They report encouraging classification performance for detecting multiple brain states and differentiating between low and high mental workloads, and anticipate the application of this approach for improving self-regulation learning.

Extant literature suggests that transcranial direct current stimulation (tDCS) improves motor learning and influences emotional and attentional processes. Soekadar et al., investigated whether tDCS induced brain activity interferes with brain self-regulation of the EEG mu-rhythm (8–15 Hz). The results of their study shows that tDCS stimulation near the C4 channel causes a signal power increase only in the lower frequencies (below 9 Hz), and hence future applications can safely combine tDCS and NF above the 9 Hz frequency range.

In another work related to tDCS, Pirulli et al., question and examine the common understanding that cathodal tDCS (c-tDCS) has an inhibitory effect on neural activity. The authors varied some important parameters of stimulation over the primary visual cortex, namely, timing, presence of pauses, duration and intensity, and tested their effect on visual orientation discrimination. In contrast to the common understanding, an improvement in task performance was observed when the c-tDCS stimulation was applied before the task for certain parameter values. The authors hypothesize that c-tDCS causes depression of cortical activity in the stimulated region but the brain reacts to restore equilibrium and this might improve visual sensitivity.

In an innovative method called motivational feedback system, Sokunbi et al., show that participants can increase or decrease BOLD levels in response to visual cues or images related to motivational processes such as hunger or craving. They present an example of visual cues of food items that grow or shrink in size, representing feedback, proportional to the subject's self-regulation of the BOLD signal in a brain region that activates hunger or craving for food.

Mathiak et al. expand the literature exploring the role of “reward” as NF signal. They compare the effect of a standard feedback (moving bars), and a social reward feedback (a smiling human face) in an fMRI-NF study that aimed to control the ACC. The experiment demonstrated a higher effectiveness of the social reward feedback, as reflected by higher ACC activity and reward-related areas (i.e., putamen). The findings also support the idea that stronger effects on ACC are achieved by social feedback compared to standard feedback during a behavioral test involving a cognitive interference task.

One major drawback of the widely used EEG-NF approach is that activity in any scalp electrode reflects a mixture of activities from multiple sources in the brain and artifacts, which may confound the actual signal from the region of interest, consequently adversely affecting learning of brain self-regulation. To circumvent this problem, White et al., implemented a real-time adaptation of the Blind Source Separation (BSS) algorithm, and tested the technique for NF training of theta oscillatory activity derived from sources in the medial temporal and parietal lobes. Pilot data demonstrate that two of four volunteers learned theta oscillatory control, suggesting moderate feasibility for the approach but calling for further research on this topic.

The second part of the current issue includes several scientific articles.

Enriquez-Geppert et al., investigate the effects of up-regulation of the frontal-midline (fm) theta power on executive function through a battery of tasks, and observed improvement in task performance in a 3-back task and reduced mixing and shifting costs in a letter/number shifting task. However, no change was observed on conflict monitoring and motor inhibition, suggesting a specific effect on proactive but not reactive mechanisms of cognitive control. Potential applications of this approach include treatment of executive dysfunctions.

In a rare investigation of the effect of reappraisal of self on emotional events in brain-damaged individuals of tumor or cyst, Falquez et al., show that legions in the right dorsolateral prefrontal cortex and the right dorsal ACC were associated with patients' impairment in the down-regulation of arousal while the intact reappraisal of healthy controls was related to increased gray matter intensity in the same regions. These results indicate that the neural and structural integrity of the right superior frontal gyrus are related to emotional regulation by reappraisal of self. The use of this approach could prove beneficial in regulation of emotional arousal.

The last part of this special issue includes scientific articles that explore the effect of brain self-regulation in symptom alleviation, a topic of great interest in the field (Ruiz et al., 2013; Buyukturkoglu et al., 2015).

Chronic pain, a condition targeted by real-time fMRI-NF since its first implantations on clinical populations, is explored in two articles of this issue. Emmert et al., investigate modulation of pain perception in a group of healthy individuals by down-regulation of anterior cingulate and anterior insula during pain stimulation, suggesting that both regions are suitable targets for reducing pain with fMRI-NF.

In a novel approach, Rance et al. (2014) move away from single-ROI self-regulation and explore the capability of individuals to increase the difference in BOLD activation of ACC and posterior insula, two regions of the pain processing network, separately targeted in previous fMRI-NF experiments and pain modulation. Although no correlation was found with pain perception, the finding that individuals can control the differential activation of two functionally connected areas is of importance for clinical applications in disorders of abnormal neural connectivity.

Regarding psychiatric disorders, Cordes et al., investigated the neural strategies used to achieve control of the BOLD signal in the ACC in schizophrenia. The results suggest that schizophrenia patients use different cognitive and neural strategies for self- regulation compared to healthy individuals. In fact, schizophrenia patients activated the dorsal subdivision of the ACC as compared to a control group of healthy individuals, which activated the rostral subdivision, giving support to the idea of a subdivision dysbalance in ACC as part of the psychopathology of the disease.

Escolano et al., explore whether cognitive deficits in depression can be alleviated by NF training of alpha power of parieto-occipital EEG signals. Patients suffering from major depression improved working memory and processing speed compared with control depression subjects (that did not received NF). An interesting outcome involving the correlation of beta power in the genual ACC correlating with processing speed suggests a role of this area in cognitive processing.

In a rare study on brain self-regulation in severe personality disorders, Sitaram et al., investigate the effect of the modulation of the brain fear circuitry in criminal psychopaths. The subjects displayed a low-success rate in regulating insula (different as compared to previous studied in healthy individuals), and a correlation between the severity of psychopathy traits and the difficulties regulating insula cortex. Interestingly, functional connectivity changes in the emotional network are observed throughout NF training, opening interesting questions on potential brain remodeling in severe personality disorders.

In another work on self-regulation of emotional brain areas, Zilverstand et al., presented individuals suffering from spider phobia with a novel dual visual feedback based on the BOLD activity in the insula pertaining to sustained anxiety and the dorsolateral prefrontal cortex pertaining to engagement in regulation. Participants of the NF group achieved down-regulation of insula activation levels by cognitive reappraisal and exhibited lower anxiety levels than the control group.

Gharabaghi et al., evaluated the advantages of EEG-NF from the epidural space in a patient with a large ischemic stroke, showing that this methodology can lead to self-regulation of sensorimotor oscillations, even when standard EEG-NF is unsuccessful.

In an effort to develop novel approaches for symptom alleviation in mood disorders, Ray et al., develop a BCI system based on a pattern classifier of brain's affective states. A subject-independent classifier was first created based on the method of Common Spatial Patterns (CSPs) from the EEG data of several healthy individuals. The BCI system then used the classifier to provide real-time NF for individuals to learn to “match” the affective states provided by the classifier. The authors anticipate the application of this approach in correcting the abnormal affective states in patients suffering from mood disorders.

Scheinost et al., attempted to identify brain connectivity patterns associated with behavioral changes due to fMRI-NF training in Obsessive Compulsive Disorder patients. In this pilot study, it is demonstrated that whole-brain connectivity in the orbitofrontaland anterior prefrontal cortex, collected from resting state-fMRI before the training, correlates with symptom alleviation following NF.

This special issue on self-regulation of the brain of emotion and attention using NF approaches interested authors to report technical and methodological advance, scientific investigations in understanding the relation between brain activity and behavior using NF, and finally studies developing clinical treatment of emotional and attentional disorders. The editors of this special issue anticipate rapid developments in this emerging field.

## Author contributions

All authors listed, have made substantial, direct and intellectual contribution to the work, and approved it for publication.

### Conflict of interest statement

The authors declare that the research was conducted in the absence of any commercial or financial relationships that could be construed as a potential conflict of interest.
